# Food-based dietary guidelines beyond language: context, food security, nutrition, and sustainability

**DOI:** 10.1016/j.lana.2026.101648

**Published:** 2026-09-22

**Authors:** Israel Rios-Castillo, Rebecca Kanter

**Affiliations:** a Food and Agriculture Organization of the United Nations, Subregional Office for Mesoamerica, Panama City, Panama; b Department of Nutrition, Faculty of Medicine, University of Chile, Santiago, Chile

We read with interest the article by Bertini and colleagues that applies an innovative natural language processing (NLP) approach to analyze dairy-related recommendations in food-based dietary guidelines (FBDGs) across 98 countries.^1^ Nevertheless, aspects of their findings deserve careful consideration.

First, the associations between lexical diversity, semantic complexity and policy development indicators should be interpreted cautiously. FBDGs are evidence-based public communication tools with actionable messages for general populations.^2^ Simpler language is deliberate to ensure accessibility, comprehension, and relevance. Globally, FBDGs must be adapted to diverse territories.^3^^,^^4^

Second, the authors acknowledge that the availability and comprehensiveness of FBDGs varies widely. These disparities stem from differences in development and dissemination, including FBDGs not publicly available. Variations in length, structure or accessibility may bias NLP-derived metrics falsely appearing as differences in dietary guidance sophistication.

Third, while NLP is efficient in analyzing large volumes of text, it cannot fully capture context in which FBDGs are developed. Such guidelines are embedded within complex food systems with sub-national differences from agricultural production to cultural food practices. General recommendations related to dairy consumption obfuscate such contextual realities including livestock production, refrigeration, and dairy product affordability. Thus, differences in linguistic complexity should avoid being interpreted as direct indicators of the appropriateness, effectiveness, or quality of FBDGs.

A fourth concern is the exclusive focus on dairy products. FBDGs should promote overall healthy diets to achieve nutritional adequacy, balance, diversity, and moderation across all food groups. Focus on a single food group provides an impartial view of how to achieve healthy diets.^4^ Dietary guidance favors broad recommendations over food-specific messages. This issue is of greater concern as study support was provided by the Scientific Committee of Dairy Products of the Chilean Dairy Consortium. Although the authors declare no competing interests, further discussion regarding the role of this collaboration would strengthen transparency and confidence in the independence of the analysis.

Finally, the interpretation of the findings should consider the global syndemic where undernutrition, climate change and overweight coexist.^5^ Globally, policymakers are challenged to balancing nutritional adequacy, food security and chronic disease prevention simultaneously through sustainable diets.

Using NLP for nutrition policy analysis is innovative and valuable. However, computational outputs should be interpreted as complementary insights rather than proxies for guideline quality. The effectiveness of FBDGs ultimately depends not on lexical sophistication, but on the capacity to support sustainable diets within the country specific food systems for which they were designed.

## Contributors

IRC conducted the initial literature search and writing of the letter. RK revised and edited the letter. Both IRC and RK approved the final version of the letter.

## Declaration of interests

RK has no competing interests to declare. IRC as an FAO staff member, the FAO Copyright Office issues a publication license for dissemination in external media. This license will be shared once the document has been accepted for publication. The views expressed in this publication are those of the author(s) and do not necessarily reflect the views or policies of the Food and Agriculture Organization of the United Nations.
